# Comparative Bioinformatic Analysis Reveals Conserved Regions in SARS-CoV-2 Genome for RAPID Pandemic Response

**DOI:** 10.3390/ijms25115764

**Published:** 2024-05-25

**Authors:** Marcela Viviana Karpuj, D. R. Shaytov, Yonat Shemer-Avni, Michael Gideon, Zakharia M. Frenkel, Sarit Sivan

**Affiliations:** 1 Department of Biotechnology Engineering, Braude College of Engineering, Karmiel 2161002, Israel; 2 Laboratory of Clinical Virology, Soroka University Medical Center, Beer-Sheva 84101, Israel; 3 Department of Neurosurgery, Soroka Medical Center, Beer-Sheva 84101, Israel; 4 Department of Software Engineering, Braude College of Engineering, Karmiel 2161002, Israel

**Keywords:** SARS-CoV-2, bioinformatic analysis, diagnostic assay, rapid testing, conserved genomic regions, point-of-care testing

## Abstract

In the face of the SARS-CoV-2 pandemic, characterized by the virus’s rapid mutation rates, developing timely and targeted therapeutic and diagnostic interventions presents a significant challenge. This study utilizes bioinformatic analyses to pinpoint conserved genomic regions within SARS-CoV-2, offering a strategic advantage in the fight against this and future pathogens. Our approach has enabled the creation of a diagnostic assay that is not only rapid, reliable, and cost-effective but also possesses a remarkable capacity to detect a wide array of current and prospective variants with unmatched precision. The significance of our findings lies in the demonstration that focusing on these conserved genomic sequences can significantly enhance our preparedness for and response to emerging infectious diseases. By providing a blueprint for the development of versatile diagnostic tools and therapeutics, this research paves the way for a more effective global pandemic response strategy.

## 1. Introduction

The availability of a reliable, accessible, and cost-effective standardized assay is crucial for preventing and containing the spread of pathogens, particularly in the absence of ancestral data for newly identified viruses [1]. This study focused on addressing this challenge using the current coronavirus disease (COVID-19) pandemic as a case study. The agent responsible for COVID-19, severe acute respiratory syndrome coronavirus-2 (SARS-CoV-2), is an enveloped, positive-sense single-stranded RNA virus. Its genome features a 5′ terminal abundant in open reading frames responsible for encoding vital proteins necessary for virus replication, while the 3′ terminal comprises five structural proteins: spike (S), membrane (M), nucleocapsid (N), envelope (E), and hemagglutinin-esterase (HE) [2]. Molecular testing was chosen as the optimal method due to its impact on public health outcomes and viral transmission reduction. The gold-standard one-step qPCR test, targeting three genes encoding for N, S, and E proteins, is currently used for SARS-CoV-2 detection [3,4,5,6]. The N protein represents the nucleocapsid of the virus, the S protein is part of the spike glycoprotein, and the E denotes the viral envelope [7,8,9]. The joint global effort to eradicate the recent SARS-CoV-2 pandemic provided vast information regarding the evolution of its variants [10,11]. Aggregated information revealed that the regions used in the gold-standard test are frequently mutated, and therefore, for some variants, these qPCR tests may lead to false-negative results [12]. Moreover, it was recently demonstrated that these regions have a high similarity to other related species such as the *severe acute respiratory syndrome coronavirus (SARS-CoV), Middle East respiratory syndrome coronavirus (MERS-CoV), human coronavirus HKU1 (hCoV-HKU1), human coronavirus OC43 (hCOV-OC43)* [13]. This might lead to the wrong diagnosis and inaccurate treatment. For these reasons, currently available genomic tests require three different sets of primers, which are updated occasionally, due to the high frequency of mutations that are often identified [4]. Also, current tests involve a decontamination process of heating the sample for 20 min at 70 °C. This has been proven to harm RNA integrity and therefore lead to false-negative results [14]. Furthermore, it is important to note that these tests rely on a minute portion of the worldwide existing variants, as they are likely to represent sequences in countries that are economically and technologically more advanced [11]. We thus predict that many more subvariants already exist, and others will naturally evolve in the future [12]. We hypothesized that the key to the development of a generic, yet specific and accurate test, which can detect current and future unknown variants, is to identify conserved regions within emerging pathogens. To this aim, using a bioinformatic-driven strategy, we identified three independent conserved regions, which are highly specific to the SARS-CoV-2 genome. In this study, we focused on a single primer set (comprising three independent primers) to develop a specific, saliva-based qPCR test which further concentrates the sample and has additional benefits that will be further discussed. Our proposed single primer set demonstrated similar results compared to the gold-standard test, which uses three independent primer sets from non-conserved regions, when starting from the same isolated RNA sample. Our data, in combination with a website reporting the various strains present in Israel [10], indicate that we detected Alpha, Beta, and Omicron. This also correlates with our in silico predictions. Most importantly, we observed no false positives, indicating that our selected primer SET1 was highly selective, as predicted by in silico analysis against SARS-CoV-1, MERS, and influenza viral genomes. Preliminary data indicate that the inclusion of a concentrating step of the total nucleic acids from a saliva sample gives better results than the gold-standard test. We present a case study of an asymptomatic individual who returned to Israel from the USA. This individual’s test results were borderline with the gold-standard Seegene method at the airport, and thus, no quarantine was required. However, upon using our new method of RNA saliva isolation in combination with a high-sensitivity qPCR assay, the individual was found to be significantly infectious. 

This prevented further transmission to family members, as the individual was advised to self-quarantine. In addition, the ability to target drugs to these conserved regions was validated with an independent recent study [15]. Early and quick detection could be crucial. In addition, the impact of COVID-19 may extend beyond pulmonary involvement, resulting in thrombotic complications, myocardial dysfunction, arrhythmias, acute coronary syndromes, acute kidney injury, gastrointestinal manifestations, hepatocellular injury, hyperglycemia with ketosis, neurological conditions, ocular symptoms, and dermatological complications [16]. Given the susceptibility of certain populations to these complications, early testing is paramount to curbing further transmission within these vulnerable groups.

In the future, an increase in sensitivity can be achieved by including additional sets of primers from the additional two conserved regions that we identified. The proposed saliva test enables self-sampling and incorporates a built-in deactivation mechanism. This eliminates the need to visit a medical facility, as the inactivated sample can be sent directly to the collection center. This approach also removes the requirement for specialized laboratories, further simplifying the process. 

## 2. Results

### 2.1. Comparative Analysis of Whole-Genome Data Using Bioinformatic Tools Revealed Conserved Regions of SARS-CoV-2

**Figure 1:**
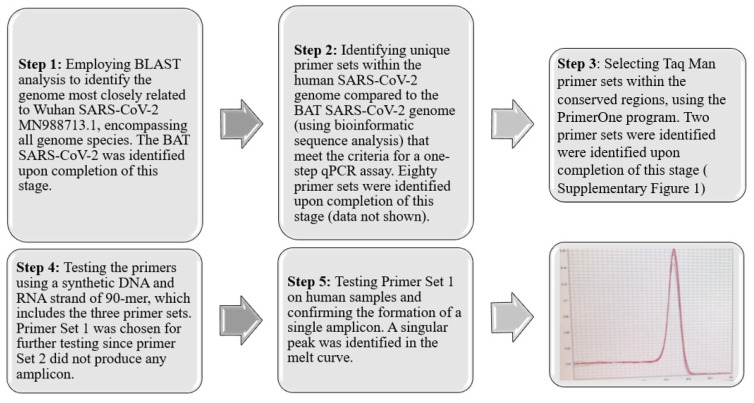
Five-Step Strategy for Identification and Primer Set Development. This strategy involves a comprehensive process of sequence analysis, verification, and refinement, culminating in the identification of an optimal primer set (SET1) that produces a specific single amplicon. This streamlined approach ensures the accurate detection of conserved regions within SARS-CoV-2. The red line represents the melting point curve indicating a single amplicon.

To develop diagnostic and therapeutic tools that can effectively combat the instability of RNA pathogens and the survival mechanisms of drug-resistant pathogens [17,18,19,20,21], it is essential to identify and target conserved regions within emerging pathogens. This strategy facilitates the detection and development of drugs against both existing and future, yet unidentified variants. In pursuit of this objective, we have devised a five-step strategy, depicted in Figure 1. The process begins with segmenting the entire COVID-19 genome (MN988713.1) into segments of 20 nucleotides, referred to as K-mers. Subsequently, certain sequences were excluded from these K-mers based on specific criteria. Initially, K-mers that occurred more than once within the COVID-19 genome were discarded. Furthermore, sequences found in the human genome or those identified in closely related species were also removed. The determination of related species was conducted by using the Basic Local Alignment Search Tool (BLAST) analysis, considering species with an identity exceeding 99% and a bit-score above 54,998 as identical. This procedure led to the isolation of K-mers uniquely associated with human COVID-19. Following this, we applied optimal qPCR criteria to the identified K-mers to select primer sets capable of generating the most effective amplicons. The criteria implemented were as follows: the CG content of the primers should range from 40% to 60%, the last five bases of the primers should include at least two G/C bases, and the ultimate nucleotide at the 3′ end should be a G or C base. Repetitive sequences such as ATATAT or ACCCC, complementary K-mers, and those prone to forming hairpins were excluded. To ensure amplification stability, the melting temperature (Tm) of the two primers was required to not differ by more than 3 °C. Additionally, the gap between the forward and reverse primers was mandated to be between 150 and 300 base pairs.

### 2.2. Data Availability

The data supporting this study are available within the article and its Supplementary Materials, including the digital data underlying the figures. Additional raw data supporting this study can be obtained from the corresponding author upon a reasonable request.

### 2.3. Code Availability

The code used to identify the conserved regions within a specific genome template is available at https://drive.google.com/drive/folders/1SAyBSMayVXR9vSPX9SiA0lGwcTfDO-4I?usp=sharing (accessed on 12 March 2024).

**Figure 2:**
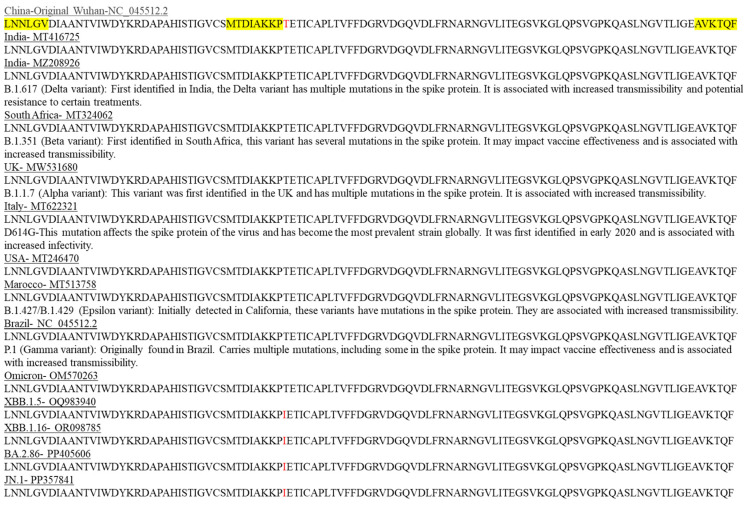
Locations of known mutations in SARS-CoV-2 protein variants are unique and are not located within the conserved regions targeted by SET1 primers. In silico verification was conducted to confirm the presence and localization of the SET1 primers (highlighted in yellow) within the protein sequences of selected SARS-CoV-2 variants. A single mutation that differs from the original Wuhan SARS-CoV-2 strain is indicated in red.

Using BLAST analysis, we validated the presence of the final three primers of SET1 in all globally known distributed SARS-CoV-2 variants. It is important to note that all three primers (highlighted in yellow in Figure 2) comprising SET1 belong to the coding region and are present in all different SARS-CoV-2 variants since the initial Wuhan SARS-CoV-2 outbreak, including recent variants.

Interestingly, there are only two mutations in this conserved region, with one mutation at the protein level occurring in the latest strains of SARS-CoV-2 (highlighted in red in Figure 2), and the second is a silent mutation. Notably, these mutation locations are not included in our selected conserved primer regions (highlighted in yellow in Figure 2 and nucleic acid sequences alignment in Supplementary Figure S6).

### 2.4. Evaluation of SET1 Primer Selectivity and Optimization of One-Step RT-qPCR Assay Using Synthetic DNA and RNA

Based on published sequences and using BLAST analysis, none of the following pathogens—*hepatitis C* [22,23], *HIV1* [24,25], *influenza A* [26,27], *influenza B* [28,29], *polio* [30,31]—contain the specific regions targeted by SET1 primers. While the reverse primer of SET1 was found to be present in *influenza B* [28,29] and partially present in MERS [32,33] and SARS [34], it is important to note that a PCR product can only be formed if both the forward and reverse primers anneal to the same template. Therefore, the SET1 primers remain specific and unique to SARS-CoV-2, and we predicted that our SET1 primers will not lead to false-positive results which is indeed the case (see figures below and Supplementary Figures S2 and S3).

The next step was to evaluate the ability of SET1 primers to produce a single amplicon, which is an essential requirement when establishing a reliable qPCR assay. Initially, the primers were tested on a synthetic DNA fragment. To minimize the cost of the synthetic DNA, we ordered a 90 bp DNA length identical to the SARS-CoV-2 genome. This fragment included the homologous sequence of the primer sequences flanking the entire amplicon and the internal primer probe sequence, excluding irrelevant nucleotide sequences within this region.

**Figure 3:**
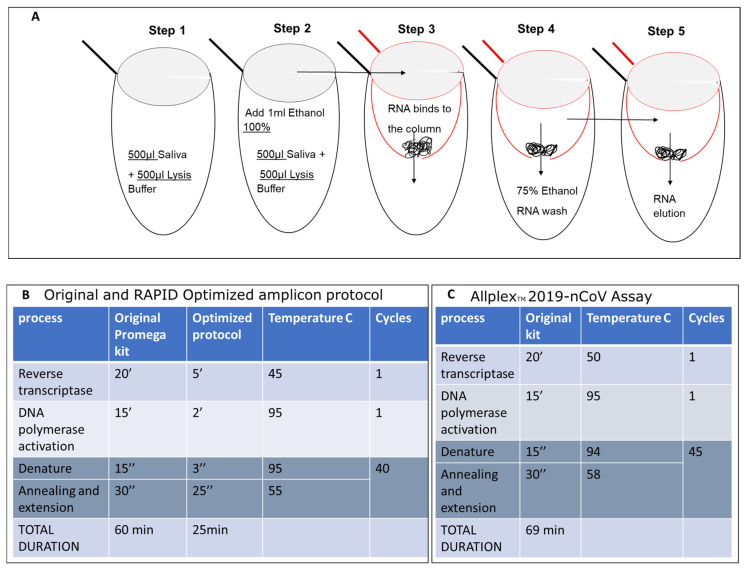
Isolation of SARS-CoV-2 Nucleic Acid from Saliva and Optimized qPCR Assay protocol. (**A**) RNA samples were isolated from 500 microliters of saliva from COVID-19-infected individuals or symptomatic patients. These samples were then mixed with an equal volume of lysis buffer (500 microliters, as shown in Figure 3A—step 1) and 1 milliliter of 100% ethanol (EtOH) (step 2). The mixture was inverted, vortexed, and applied to a preprepared glass wool column (step 3), followed by centrifugation at maximum speed, processing five aliquots of 400 microliters each. The flow-through was discarded. The column was then placed in a new 2 mL test tube and washed twice with 500 mL of 75% EtOH (step 4). RNA was eluted in double-distilled water (18 microliters of DDW preheated to 64 °C) and directly used in the one-step qPCR. Steps 1 and 2 are carried out in the same 2 mL tube. The mixture was transferred into a 0.5 mL tube (red tube), which has a hole made using a 21 G needle (3.81 cm), and placed into a new 2 mL tube (black tube) during loading. The elution of RNA was performed into a new 1.5 mL Eppendorf RNase-free tube. The original Promega one-step protocol was adapted to suit the shortened amplicon produced by SET1 primers. This includes the melting point based on the primer sequences and the elongation time based on the length of the predicted amplicon (**B**) and compared with the original Allplex Seegen protocol (**C**).

This was followed by the development of a one-step qPCR assay using a synthetic RNA fragment employing the GoTaq^®^ Probe 1-Step RT-qPCR System from Promega (Catalog # A6120 and A6121). It is important to note that our *in-silico* analysis (illustrated in Figure 1, steps 1–3) resulted in three possible targets. However, by selecting a primer set located closer to the 3′ end (Supplementary Figure S1B, SET1 primer), we were able to decrease the duration of the one-step RT-qPCR process, resulting in a reduced incubation time for the cDNA synthesis step from 35 to 7 min and shortening the amplification cycles from 26 to 18 min (Figure 3B). This reduced the overall qPCR process to 25 min for 40 cycles. It is important to note that people with a high titer, who are often symptomatic, mostly test positive with Cts of 23–28, suggesting that results will be obtained within 17–20 min using our results, as compared to the gold-standard test which takes approximately two hours.

### 2.5. Evaluation of SET1 Primers Using the Shorter RAPID One-Step RT-qPCR Protocol on Nucleic Acid Isolated from Saliva

**Figure 4:**
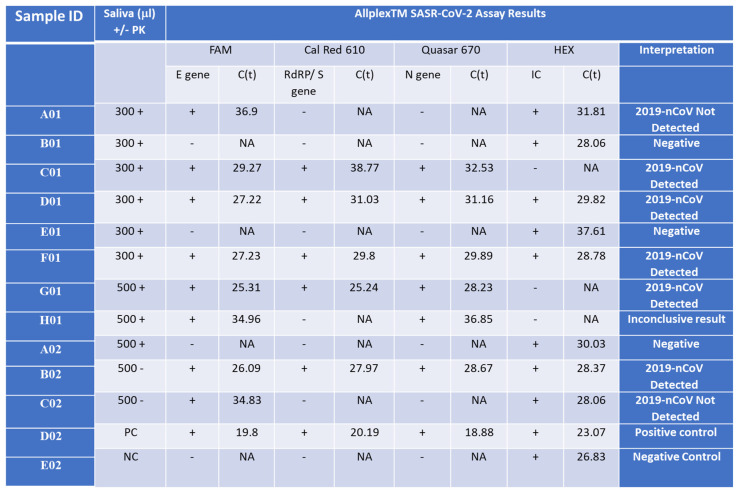
Detection of the Wuhan SARS-CoV-2 in saliva samples from infected patients. Saliva samples (300 or 500 µL) were collected from SARS-CoV-2 patients with Ct lower than 34 for the E gene (C01, D01, F01, G01 = B02), a SARS-CoV-2 patient with Ct higher than 34 or the E gene (A01 = H01 = C02), and control patients who represent normal and symptomatic flu-like patients who were negative with the Seegene test (B01, E01, A02) and incubated with (+PK) or without (-PK) proteinase K. D02 and E02 are positive and negative control (PC and NC), respectively, provided by the Seegen Allplex TM assay. The Seegene gold-standard protocol involves four different primer sets, each targeting specific regions (E, S, and N genes) of the SARS-CoV-2 viral genome, along with a fourth primer set for a spiked positive control sample (HEX). A qPCR assay with no template was used as a negative control (NC) for the qPCR assay. The interpretation column indicates the call by the gold-standard Seegene platform. ‘NA’ stands for ‘not applicable’, indicating no detected CT value. ‘2019-nCoV Detected’ denotes the declaration of a SARS-CoV-2-positive sample. ‘Negative’ indicates a clear-cut negative sample. ‘2019-nCoV Not Detected’ refers to a borderline sample. ‘Positive control’ denotes an internal positive control amplicon sample provided by the manufacturer, while ‘Negative control’ indicates no template.

To further test our ability to detect and amplify RNA from saliva, we first spiked 90 bp of synthetic RNA, partially coding for the Wuhan SARS-CoV-2 genome as previously described, in saliva collected from uninfected individuals. Each saliva sample (500 µL) was mixed with an identical volume of lysis buffer in a 2 mL tube. This process exposes the viral genome while preserving its integrity, which is crucial for accurate testing and avoiding false negatives in RNA viruses. Subsequently, 1 mL of 100% ethanol was added to the same 2 mL tube and mixed with the lysate to prepare the nucleic acid for binding to the glass column. Glass wool, known for its net positive charge and hydrophobic properties (Carlo Erba Cat# 65997-17-3 batch V71674097L), was packed into a 0.5 mL Eppendorf tube (Figure 3A) with a hole at the bottom created by a needle with a gauge of 21. This 0.5 mL tube was then placed into a 2 mL collecting tube. The mixture was passed through the column several times using a benchtop centrifuge, with each round of centrifugation at 10,000× *g* for 1 min. This process enabled the processing of large volumes without preconditioning of the column. The column was washed twice in the same manner with 75% ethanol, and then the nucleic acid was eluted into a new Eppendorf tube using 18 µL of preheated double-distilled water (DDW) at 65 °C. Thereafter, 2.3 µL of eluted nucleic acid was added into the qPCR mixture as previously described. This protocol resulted in a single amplicon using our RAPID one-step qPCR method. In collaboration with Soroka Medical Center, we initially assessed our capability to concentrate SARS-CoV-2 RNA from saliva using the established protocol alongside the gold-standard Seegene one-step qPCR assay (Figure 4). 

We investigated the addition of proteinase K (PK) to the lysis buffer but found that the Ct values of samples processed with or without PK were similar. Therefore, we decided to exclude PK from the isolation process for future samples. Upon comparing the sensitivity of isolated samples using the Seegene swab tests to those isolated with our columns and subsequently detected using the Seegene gold-standard protocol, our assay exhibited a slight reduction in sensitivity by 2–4 cycles in both scenarios (Supplementary Figure S5). Remarkably, the detection of the E gene was consistently less sensitive than that of all other primer sets in the gold-standard assay. Negative control samples tested negative for the assay using both protocols (Figure 4 B01, E01, A02, E02). However, in some instances, internal primer amplification (HEX) appeared to be inhibited using our nucleic acid isolation protocol (Figure 4 C01, G01, H01). It is noteworthy that before the optimization of our qPCR RAPID test, a sample with a higher Ct value than 34 for the E gene was not detected or provided inconclusive results from saliva (samples A01 = H01 = C02), regardless of the initial saliva volume processed or the presence of PK in the lysis buffer.

### 2.6. Validation of the Shortened RAPID One-Step qPCR Assay Using Various Positive and Negative Samples

The culmination of our study involved validating the optimized assay by comparing it with a variety of samples, both with low and high cycle threshold (Ct) values, isolated using the gold-standard Seegene test, and concurrently testing them with our RAPID assay. Our findings affirm that our test is as accurate as the gold standard Seegene test, successfully detecting all three variants assessed (Delta variant—Figure 4, Supplementary Figures S2 and S5, the Beta variant—Supplementary Figure S3, and the Omicron variant—see figure below). However, despite processing samples using the gold-standard isolation method with the Seegene STARlet equipment following the manufacturer’s protocol, and then testing them with our qPCR assay (Supplementary Figure S2), no false positives were detected. Nonetheless, the detection sensitivity for each sample was lower. This could be attributed to the samples no longer being as fresh, as RNA samples are relatively sensitive and may degrade over time, leading to a decrease in the number of detectable copies.

### 2.7. Optimization and Significant Reduction in Time of the One-Step qPCR Method, Enhancing Sensitivity for Future SARS-CoV-2 Variants

**Figure 5:**
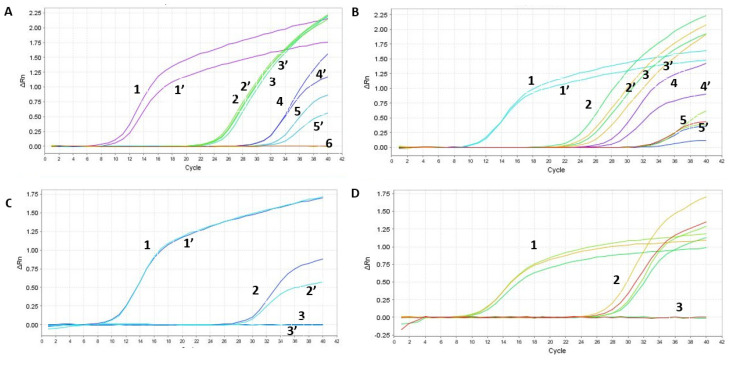

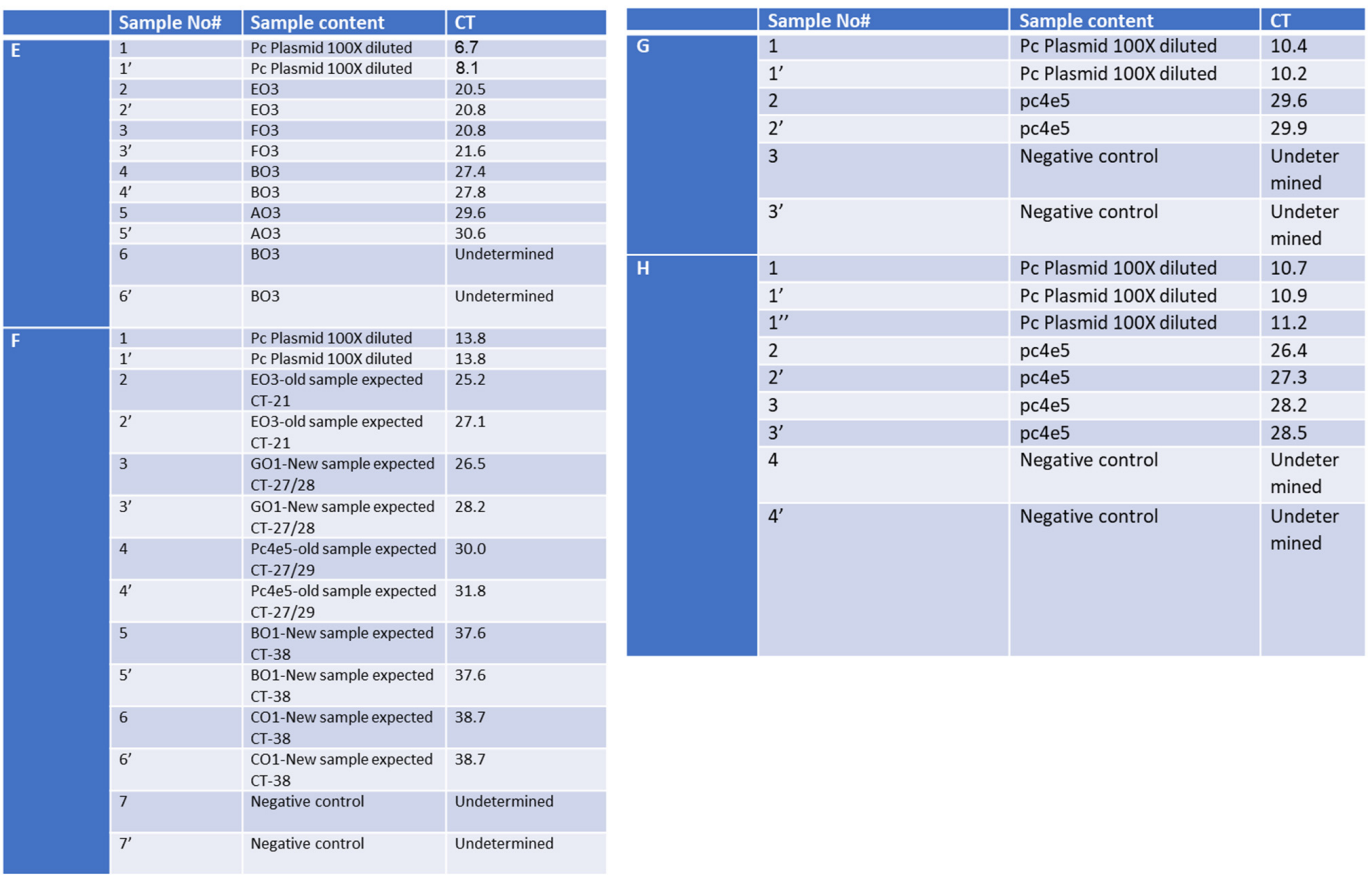
Optimization of the RAPID one-step qPCR reagents using SET1 primers on human RNA isolated using the gold-standard Seegene protocol. Graph **A and Table E**—enzyme comparison, Graph **B and Table F**—new and old plate from Soroka Hospital, Graph **C and Table G**—primer dilution, Graph **D and Table H**—testing new primer mixture. The detailed results, representing each graph (**A**–**D**), are depicted in (**A**–**D**), respectively. ‘PC’ stands for positive control and denotes a plasmid containing the specific SET1 amplicon. Negative control denotes no nucleic acid input. ‘Undetermined’ indicates a clear-cut negative sample reading by the step-one qPCR machine. Table E contains the samples described in Graph A, Table F describes the samples in Graph B, Table G describes the samples in Graph C, and Table H describes the samples in Graph D.

Since in some cases we had equal or higher Cts (Supplementary Figure S2), we worked further on the optimization of the column isolation step as well as on the one-step qPCR assay. First, we optimized the reagents using our SET1 primer set. The tested parameters included: the comparison of the one-step qPCRenzyme mix used 10 times (samples 1–6 in Figure 5A) to a new enzyme batch (samples 1′–6′ in Figure 5A); fresh (samples 1, 1′, 3, 3′ in Figure 5B) versus frozen RNA samples from Soroka Hospital (samples 2, 2′, 4, 4′, 5, 5′, 6, 6′ in Figure 5B); two different primer dilutions (0.2 µL of the primer mixture for samples 1–3 and 0.5 µL for samples 1′–3′ in Figure 5C); and comparing an old batch of primer mixture from IDT (samples 1–4) to a new batch (samples 1′–4′ in Figure 5D). Our findings show that cycling the enzyme between −80 °C storage and ice up to 10 times does not affect the activity of the reverse transcriptase or DNA polymerase, as evidenced by the identical sensitivity of both sample sets. Furthermore, our analysis indicates that frozen RNA samples, isolated using the Seegene method, exhibited a loss in sensitivity, as opposed to freshly isolated samples. Consequently, this manuscript only presents results from freshly isolated samples, using the optimal primer mixture dilution. We explored different concentrations of magnesium chloride in the PCR mixture but found the kit’s recommended concentration was optimal, hence no adjustments were made to this parameter.

Given that our assay targets a single short amplicon of 302 bp, full cDNA amplification of the pathogen was unnecessary. As a result, we substantially shortened the reverse transcription (RT) step from the standard 20 min protocol to 5 min at 45 °C, followed by a reduction in DNA polymerase activation time from 15 min to 2 min at 95 °C. We opted for a concise qPCR amplification protocol, reducing the denaturation step at 95 °C from 15 s to 3 s. The annealing and extension steps were combined at 55 °C, reducing the overall duration from 30 s to 25 s. The entire process was completed within 25.67 min, significantly shorter than the duration using the standard qPCR assay (Figure 5B). The reagents for our qPCR process included 5 µL CRX Mix Buffer, 0.5 µL primer set 1, 0.2 µL enzyme, 1.1 µL DDW, with 3.2 µL of the unknown sample, 3.2 µL of DDW for the negative control, or 3.2 µL of the SARS-CoV-2 qPCR product for the positive control. This represents a 50% reduction in material use, and hence in respective costs, compared to the Seegene Allplex protocol (Figure 5C).

### 2.8. Validation of the Optimized Assay Using Positive Samples, including a Critical Case Study

**Figure 6:**
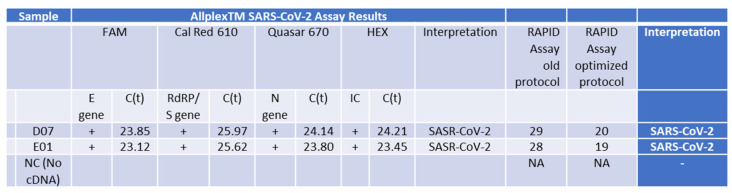
PCR Analysis of RNA Samples from Patients with the Delta Variant using the fully optimized RAPID assay protocol. This table shows results from samples isolated using the Seegene gold-standard method, tested with the standard Seegene PCR test in comparison to our RAPID test before (old protocol) optimization parameters (for details, see Figure 5) and using the improved RAPID assay fully optimized protocol.

The culmination of our study involved the comparison of RNA isolated from SARS-CoV-2-positive individuals using the Seegene isolation protocol and test to our optimized RAPID protocol. Our findings affirm that our test is as accurate as the gold-standard Seegene test, successfully detecting all three variants assessed (Delta—Figure 6 and Supplementary Figure S2, the Beta variant—Supplementary Figure S3, and the Omicron variant—Supplementary Figure S4). Furthermore, our RAPID optimized protocol significantly increased the assay sensitivity (Figure 6). 

The effectiveness of the RAPID test optimization is particularly evident when comparing Supplementary Figures S2 and S6. In addition to our consistent negative results matching the gold-standard method by Seegene, it is important to highlight that our negative control samples consisted of a combination of irrelevant samples and symptomatic cases resembling flu-like symptoms, which were not disclosed to us at any point. While we do not anticipate having all similar viruses to SARS-CoV-2 in our sample inventory, we utilized the well-established IDT PrimerQuest tool (https://eu.idtdna.com/PrimerQuest/Home/Index, accessed on 18 April 2024) to predict false-positive amplicons. Our analysis clearly demonstrates that none of these other similar viruses could produce false-positive amplicons when used as templates.

**Figure 7:**
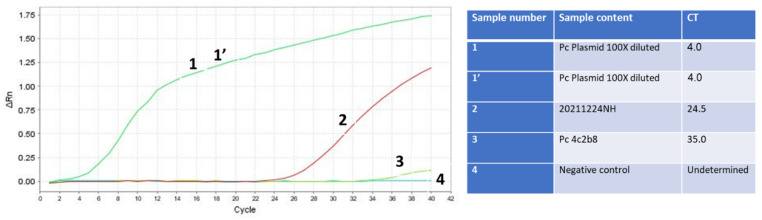
RNA isolation of the Omicron SARS-CoV-2 variant in combination with the final optimization of the RAPID test is more sensitive than the swab gold-standard Seegene test used at the Israeli airport. Samples 1 and 1’ (green line) are duplicates of the PCR product of SET1 cloned in the TOPO TA plasmid, serving as positive controls. Sample 2 (red line) is the unknown sample tested for infectivity, which was eventually confirmed to be positive for SARS-CoV-2. Sample 3 (light green line) is the SARS-CoV-2 human RNA isolated and detected using the gold standard test, also serving as a positive control. Sample 4 (dark green line) is the negative control sample with no nucleic acid template.

The conclusive test involved integrating the RNA isolation method with the optimized RAPID test, contrasting it against the gold-standard test conducted at an Israeli airport. A sample labeled 20211224NH, obtained from a US passenger who arrived in Israel and received a borderline test result at the airport, was selected for this purpose. The ambiguous result was deemed insufficient to mandate quarantine, and the individual proceeded to a family event with his 90-year-old grandmother. Utilizing our RNA isolation method and conducting the RAPID test, we unequivocally determined that the individual was infected, with a Ct value of 24 (Figure 7). It is possible that since our RAPID test does not require the decontamination process, we have more RNA template initially than the swab tests that require decontamination. This certainly reduces the assay sensitivity and could, as previously demonstrated [14], lead to false-negative results.

It is important to note that in the Seegene assay, the E primer set demonstrated the highest sensitivity across all tested samples. Our previous protocol, while shorter, was less sensitive. However, through the optimization of saliva concentration and the utilization of the one-step qPCR assay with SET1 primers, the RAPID test became significantly faster and more sensitive compared to the gold-standard test. These findings remain consistent across all variants, from the Delta to the Omicron SARS-CoV-2 variants (Figure 6 and Figure 7). Notably, the conservation of all variants within the SET1 primer region suggests that this enhanced sensitivity will endure for both current and future variants.

## 3. Discussion

Our study represents a significant advancement in the development of diagnostic tools for SARS-CoV-2. We leveraged the power of comparative bioinformatic analysis to identify conserved genomic regions within the virus, including areas that were not previously included in diagnostic tests, such as non-coding regions. Through meticulous analysis and experimental validation, we have demonstrated that focusing on these conserved regions enables the creation of a diagnostic assay that is not only rapid and cost-effective (Supplementary Figure S7) but also exhibits high sensitivity (Figure 6 and Figure 7) and specificity across a broad spectrum of SARS-CoV-2 variants (Supplementary Figures S3–S5), including those yet to emerge.

The development of our novel RAPID saliva-based qPCR test, utilizing a single primer set targeting these conserved regions, marks a pivotal advancement in the field of molecular diagnostics. This approach not only simplifies the testing process by reducing the need for multiple sets of primers but also enhances the test applicability across different settings, from clinical laboratories to point-of-care testing. The incorporation of a sample concentration step further elevates the sensitivity of our assay, making it superior to the current gold-standard tests (Figure 6). Additionally, a saliva-based test reduces the psychological barriers associated with swab tests.

The ability of our assay to detect SARS-CoV-2 in cases where gold-standard methods yield borderline results, such as the case of the asymptomatic individual presented, underscores the practical implications of our findings and further exemplifies its potential to significantly improve public health response by preventing undetected transmission.

Moreover, the specificity of our primer set, validated through extensive comparative analysis with other pathogens, ensures that our assay can distinguish SARS-CoV-2 from other viruses with high accuracy. This specificity is crucial for avoiding misdiagnosis and ensuring that patients receive the appropriate care. The optimization of the assay process, resulting in a substantial reduction in both the duration and the resources required for testing, addresses critical challenges in the pandemic response, including test availability and supply chain pressures. By streamlining the testing process, we not only make it more accessible and feasible for widespread implementation but also enhance its efficiency, enabling quicker turnaround times for results. Moreover, this strategy enabled calibration and optimization of the test in a BSL-2 laboratory setting. This approach reduced the dependency on the rare and significantly more expensive BSL-3 or BSL-4 facilities, thereby enhancing test accessibility and reducing associated costs. Also, the disease can extend its impact beyond pulmonary symptoms, potentially leading to severe thrombotic complications, myocardial dysfunction, arrhythmias, acute coronary syndromes, acute kidney injury, gastrointestinal distress, hepatocellular injury, hyperglycemia with ketosis, neurological disorders, ocular manifestations, and dermatological issues. Given the susceptibility of certain populations to these complications, early testing is paramount to reducing further transmission within these vulnerable groups.

Utilizing saliva testing offers distinct advantages over traditional swab methods. The inclusion of a decontamination solution within the collection buffer not only stabilizes RNA but also enhances convenience, making self-testing more accessible. This approach is particularly beneficial for individuals such as children or those sensitive to traditional swab tests, thereby reducing reluctance to undergo testing. By enabling self-testing, there is a decreased likelihood of further transmission, ultimately mitigating the spread of infection within susceptible populations.

The added value of such an approach lies in the identification of conserved regions that could potentially serve as specific anti-viral targets. Interestingly, recent studies have demonstrated that the infectivity of SARS-CoV-2 can be inhibited by analogs that bind to the non-structural protein-15 (NSP15), which contains a domain crucial to the viral life cycle known as uridylate-specific endoribonuclease (EndoU) [9]. This region, as confirmed by our bioinformatic approach, is known to be specific to the virus. It encompasses the domain identified as one of the conserved regions in our manuscript, which includes SET1 primers utilized in the RAPID test we have established.

## 4. Materials and Methods

After signing an informed consent, samples were obtained by swabbing the nasopharynx and oropharyngeal cavity each with a different swab using standard techniques in the same manner as previously conducted [35].

- Nucleic acid was extracted using the Seegene STARlet equipment following the manufacturer’s protocol.
- Allplex™ 2019-nCoV Assay was ordered from Seegene (Cat. No. RP10243X).

**Nucleic acid extraction** from saliva was conducted using the following protocol. Lysis buffer consisted of 0.14 M NaOH, 0.24 M NaCl, 0.4% sodium sulfite, 0.2% dithiothreitol (DTT) or β-mercaptoethanol, 0.02% SDS, 0.4% Tween 80, and 4.5 M guanidinium isothiocyanate, all dissolved in 10 mM Tris-HCl and 1 mM EDTA (TE buffer, pH 8). Elution was performed using preheated double-distilled water at 64 °C. One-step qPCR was performed using our unique primer sets in combination with the GoTaq^®^ 1-Step RT-qPCR System protocol (Promega, Cat. No. A6020). Modifications included changes in duration and temperature of each stage, and we reduced the total volume of the reaction to 10 µL. A positive plasmid was created by cloning the PCR product of the SET1 primers using the TOPO TA cloning kit for subcloning (Thermo Fisher cat# 451641). A stock of 80 ng was diluted 1:1000 with DDW and 3.2 µL was added to the qPCR reaction.

**Ordered reagents:**
  - GoTaq^®^ 1-Step RT-qPCR System (Promega, Cat. No. A6020) was used for the one-step qPCR.
  - Primer sets (Prime time^®^ Std qPCR Assay (500rXn), as well as synthetic RNA and DNA were custom ordered from Integrated DNA Technologies (IDT).

Glass wool used to create the columns was ordered from Carlo Erba (Cat. No. 457521).

## 5. Conclusions

In conclusion, our seminal study lays the foundations for a new generation of diagnostic assays that can combat the challenges posed by rapidly mutating pathogens like SARS-CoV-2 more efficiently. By focusing on conserved genomic regions, we provide a blueprint for the development of diagnostic tools that are both versatile and robust, offering a strategic advantage in the ongoing and future battles against pandemics. Our findings advocate for a paradigm shift in the design of diagnostic assays, emphasizing the importance of adaptability and foresight in pandemic preparedness and response.

## Data Availability

Data are contained within the article or Supplementary Materials.

## Supplementary Materials

The following supporting information can be downloaded at: https://www.mdpi.com/article/10.3390/ijms25115764/s1.
