# Supplementary material for: Comparative Bioinformatic Analysis Reveals Conserved Regions in SARS-CoV-2 Genome for RAPID Pandemic Response
Source: Int J Mol Sci. 2024 May 25;25(11):5764. doi: 10.3390/ijms25115764 (PMC11172030; doi:10.3390/ijms25115764)
Supplement: Supplementary file 1 [file ijms-25-05764-s001.zip › ijms-2959810-supplementary.pdf]

Comparative Bioinformatic Analysis Reveals Conserved Regions in SARS-CoV-2 Genome for RAPID Pandemic Response

Supplementary figures

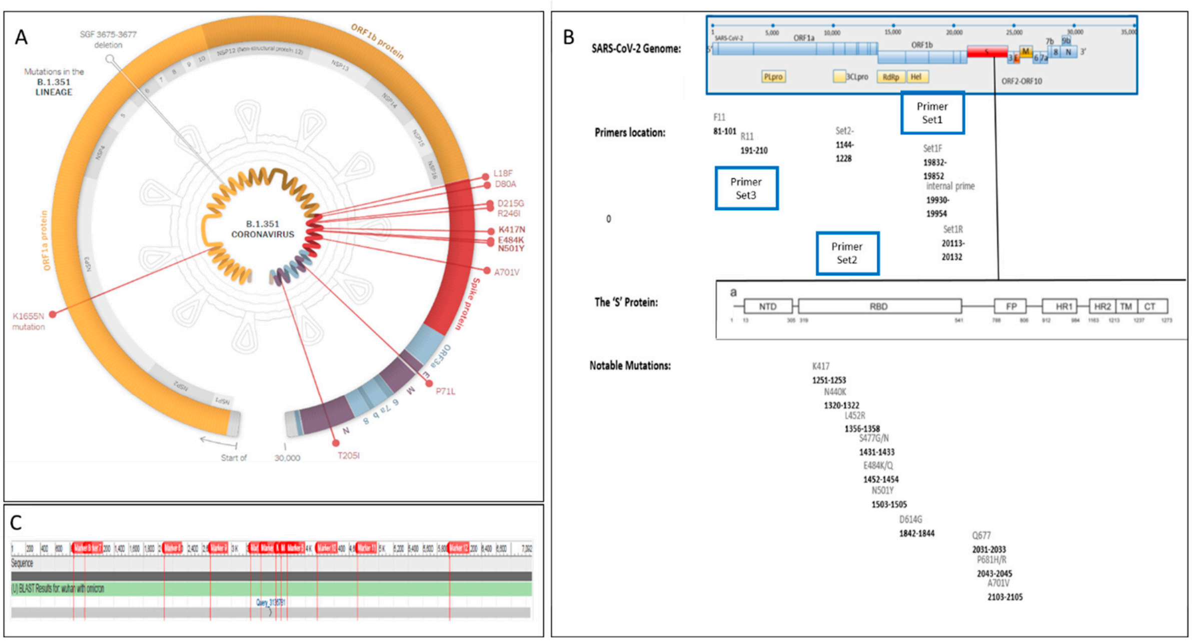

Supplementary Figure S1. The three primer sets identified in this work are conserved while the primers used in the current gold standard COVID19 tests are within regions prone to mutations. Illustration showing the position of identified mutations within the S, E, and N regions for the current gold standard test (A) compared to the three primer sets (blue rectangles) utilized in this study (B). Primer set 1, located in the conserved region of ORF1b, is present in worldwide SARS-COV-2 variants and does not overlap with any of the 13 mutations, mismatches, or deletions. This is evident from the absence of red flags at these positions when using the NCBI BLAST Tool to compare the Omicron variant with the original Wuhan SARS-Cov2 variant (C).

Sample	AllplexTM SASR-CoV-2 Assay Results									RAPID Assay Results	
	FAM		Cal Red 610		Quasar 670		HEX		Interpretatio n	C(t)	**Interpretatio n**
	E gene	C(t)	RdRP/ S gene	C(t)	N gene	C(t)	IC	C(t)			
C07	+	26.33	+	28.60	+	26.27	+	23.38	SASR-CoV-2	32	**SASR-CoV-2**
D07	+	23.85	+	25.97	+	24.14	+	24.21	SASR-CoV-2	29	**SASR-CoV-2**
E07	-	NA	-	NA	-	NA	+	23.38	-	NA	**-**
F07	-	NA	-	NA	-	NA	+	23.24	-	NA	**-**
G07	+	21.85	+	24.03	+	21.91	+	23.15	SASR-CoV-2	27	**SASR-CoV-2**
H07	+	26.83	+	29.34	+	27.23	+	23.12	SASR-CoV-2	32	**SASR-CoV-2**
A08	+	35.17	+	37.10	+	34.13	+	23.53	SASR-CoV-2	NA	**-**
B08	+	37.85	-	NA	+	33.53	+	23.59	SASR-CoV-2	NA	**-**
C08	-	NA	-	NA	-	NA	+	23.60	-	NA	**-**
D08	+	33.67	+	36.23	+	33.25	+	22.65	SASR-CoV-2	NA	**-**
E08	-	NA	-	NA	-	NA	+	23.67	-	NA	**-**
F08	-	NA	-	NA	-	NA	+	23.51	-	NA	**-**
E01	+	23.12	+	25.62	+	23.80	+	23.45	SASR-CoV-2	28	**SASR-CoV-2**
NC (no cDNA)										NA	**-**

Supplementary Figure S2. Comparative PCR Results for RNA Samples Isolated from Patients Infected with the Delta Variant. This table presents the outcomes of RNA samples isolated using the gold standard method and tested by the conventional gold standard test (SEEGENE) and our preliminary qPCR assay, before optimization.

Sample-Plate 4D1	AllplexTM SASR-CoV-2 Assay Results							RAPID Assay Results	
	FAM		Cal Red 610		Quasar 670		Interpretation	C(t)	Interpretation
	E gene	C(t)	RdRP/ S gene	C(t)	N gene	C(t)			
A1	+	17.5	+	18.4	+	17.3	SASR-CoV-2	17.5	SASR-CoV-2
B2	+	30.0	+	31.4	+	30.1	SASR-CoV-2	30.0	SASR-CoV-2
C2	+	39.0	+	39.8	+	35.3	SASR-CoV-2	39.0	SASR-CoV-2
H2	+	26.8	+	28.5	+	26.5	SASR-CoV-2	26.8	SASR-CoV-2
C3	+	35.6	+	38.6	+	36.5	SASR-CoV-2	25.6	SASR-CoV-2
D3	+	28.3	+	29.5	+	28.9	SASR-CoV-2	28.3	SASR-CoV-2
F3	+	21.8	+	23.2	+	22.3	SASR-CoV-2	21.8	SASR-CoV-2
H3	+	26.8	+	28.5	+	26.7	SASR-CoV-2	26.7	SASR-CoV-2
B3	+	30.9	+	33.2	+	31.0	SASR-CoV-2	30.9	SASR-CoV-2
F5	+	18.5	+	19.6	+	19.3	SASR-CoV-2	18.1	SASR-CoV-2
B7	+	32.8	+	33.9	+	32.2	SASR-CoV-2	32.8	SASR-CoV-2
G7	+	25.3	+	27.5	+	25.4	SASR-CoV-2	25.3	SASR-CoV-2
C7	-	NA	-	NA	-	NA	-	NA	-
C10	-	NA	-	NA	-	NA	-	NA	-
D10	-	NA	-	NA	-	NA	-	NA	-
E10	-	NA	-	NA	-	NA	-	NA	-
F10	-	NA	-	NA	-	NA	-	NA	-
G10	-	NA	-	NA	-	NA	-	NA	-
H10	-	NA	-	NA	-	NA	-	NA	-
A11	-	NA	-	NA	-	NA	-	NA	-
B11	-	NA	-	NA	-	NA	-	NA	-
C11	-	NA	-	NA	-	NA	-	NA	-
D11	-	NA	-	NA	-	NA	-	A	-

Supplementary Figure S3. PCR Analysis of RNA Samples from Patients with the Beta variant. A Comparative Analysis between the Gold Standard SEEGENE Test and the RAPID Assay. Sample A1-G7 represent SARS-CoV-2 infected patients and samples C7-D11 represent negative control. Negative control were tested negative for the test using both assays (NA).

Plate	Sample	AllplexTM SASR-CoV-2 Assay Results							RAPID Assay Results	
		FAM		Cal Red 610		Quasar 670		Interpretation	C(t)	Interpretation
		E gene	C(t)	RdRP/ S gene	C(t)	N gene	C(t)			
4B1A	A01	-	NA	-	NA	-	NA	-	NA	-
	B01	-	NA	-	NA	-	NA	-	NA	-
	C01	-	NA	-	NA	-	NA	-	NA	-
	D01	-	NA	-	NA	-	NA	-	NA	-
	F01	-	NA	-	NA	-	NA	-	NA	-
4B1C	C01	+	24	+	27	+	27	SASR-CoV-2	28	SASR-CoV-2
	A02	+	25	+	28	+	28	SASR-CoV-2	29	SASR-CoV-2
	B02	+	24	+	26	+	27	SASR-CoV-2	28	SASR-CoV-2
	C02	+	20	+	23	+	22	SASR-CoV-2	24	SASR-CoV-2
	E02	+	20	+	23	+	23	SASR-CoV-2	25	SASR-CoV-2
	F02	+	22	+	24	+	24	SASR-CoV-2	26	SASR-CoV-2
	A03	+	14	+	17	+	17	SASR-CoV-2	18	SASR-CoV-2
	B03	+	19	+	22	+	22	SASR-CoV-2	23	SASR-CoV-2
	E03	+	20	+	23	+	22	SASR-CoV-2	25	SASR-CoV-2
	G03	+	15	+	18	+	18	SASR-CoV-2	19	SASR-CoV-2
	F03	+	19	+	21	+	21	SASR-CoV-2	23	SASR-CoV-2
	E03	+	18	+	21	+	21	SASR-CoV-2	22	SASR-CoV-2

Supplementary Figure S4. PCR Analysis of Omicron Variant Samples: Comparing the Gold Standard SEEGENE Test with the RAPID Assay.

Sample Number	E-gene RAPID	E-gene Seegene	RdRP-gene RAPID	RdRP-gene Seegene	N-gene RAPID	N-gene Seegene	Internal Control RAPID	Internal Control Seegene	Seegene Call For RAPID
A01	36.91		NA		NA		31.81		COVID-19
A01	NA		NA		NA		28.06		Negative
B01	29.27	NA	38.77	32.45	32.53	33.55	NA	25.95	COVID-19
C01	27.22	25.47	31.03	26.6	31.16	26.95	29.82	25.96	COVID-19
D01	NA	NA	NA	37.05	NA	37.77	37.61	25.68	Negative
E01	27.24	25.78	29.8	27.9	29.89	27.38	28.78	26.43	COVID-19
F01	25.31	23.59	26.24	24.87	28.23	26.35	NA	26.8	COVID-19
G01	34.96	NA	NA	33.89	36.85	33.82	Na	27.04	COVID-19
H01	NA	NA	NA	NA	NA	36.95	30.03	25.7	Negative
A02	26.09		29.97		28.67		28.37		COVID-19
B02	34.83		NA		NA		28.06		COVID-19
Positive control	19.8		20.19		18.88		23.07		COVID-19
Negative control	NA		NA		NA		26.83		Negative

Supplementary Figure S5. Clinical samples comparing saliva and swab tests. Samples 1-6 are saliva samples 2 control 4 COVID-19 positive. Three COVID-19 genes for the open reading frame of the COVID-19 E, RdRp and N protein are tested in the Seengen RT-PCR validated Kit. ORT-samples which were isolated with our recently developed protocol and in parallel some samples have been isolated with the Seegen protocol.

<u>China-Original Wuhan-NC_045512.2</u>

TACTCAATAATTTGGGTGTGGACATTGCTGCTAATACTGTGATCTGGGA
CTACAAAAGAGATGCTCCAGCACATATATCTACTATTGGTGTTTGTTCTATGACTGACATAGCCAAGAAA
CCAACTGAAACGATTTGTGCACCACTCACTGTCTTTTTTGATGGTAGAGTTGATGGTCAAGTAGACTTAT
TTAGAAATGCCCGTAATGGTGTTCTTATTACAGAAGGTAGTGTTAAAGGTTTACAACCATCTGTAGGTCC
CAAACAAGCTAGTCTTAATGGAGTCACATTAATTGGAGAAGC

<u>India- MT416725</u>

TACTCAATAATTTGGGTGTGGACATTGCTGCTAATACTGTGATCTGGGA
CTACAAAAGAGATGCTCCAGCACATATATCTACTATTGGTGTTTGTTCTATGACTGACATAGCCAAGAAA
CCAACTGAAACGATTTGTGCACCACTCACTGTCTTTTTTGATGGTAGAGTTGATGGTCAAGTAGACTTAT
TTAGAAATGCCCGTAATGGTGTTCTTATTACAGAAGGTAGTGTTAAAGGTTTACAACCATCTGTAGGTCC
CAAACAAGCTAGTCTTAATGGAGTCACATTAATTGGAGAAGC

<u>India- MZ208926</u>

TACTCAATAATTTGGGTGTGGACATTGCTGCTAATACTGTGATCTGGGA
CTACAAAAGAGATGCTCCAGCACATATATCTACTATTGGTGTTTGTTCTATGACTGACATAGCCAAGAAA
CCAACTGAAACGATTTGTGCACCACTCACTGTCTTTTTTGATGGTAGAGTTGATGGTCAAGTAGACTTAT
TTAGAAATGCCCGTAATGGTGTTCTTATTACAGAAGGTAGTGTTAAAGGTTTACAACCATCTGTAGGTCC
CAAACAAGCTAGTCTTAATGGAGTCACATTAATTGGAGAAGC

B.1.617 (Delta variant):

TACTCAATAATTTGGGTGTGGACATTGCTGCTAATACTGTGATCTGGGA
CTACAAAAGAGATGCTCCAGCACATATATCTNNNNNNNNNNNNNNNNNNNNNNNNNNTAGCCAAGAAA

CCAACTGAAACGATTTGTGCACCACTCACTGTCTTTTTGATGGTAGAGTTGATGGTCAAGTAGACTTAT
TTAGAAATGCCCGTAATGGTGTTCTTATTACAGAAGGTAGTGTTAAAGGTTTACAACCATCTGTAGGTCC
CAAACAAGCTAGTCTTAATGGAGTCACATTAATTGGAGAAGC

South Africa- MT324062

TACTCAATAATTTGGGTGTGGACATTGCTGCTAATACTGTGATCTGGGA
CTACAAAAGAGATGCTCCAGCACATATATCTACTATTGGTGTTTGTTCTATGACTGACATAGCCAAGAAA
CCAACTGAAACGATTTGTGCACCACTCACTGTCTTTTTGATGGTAGAGTTGATGGTCAAGTAGACTTAT
TTAGAAATGCCCGTAATGGTGTTCTTATTACAGAAGGTAGTGTTAAAGGTTTACAACCATCTGTAGGTCC
CAAACAAGCTAGTCTTAATGGAGTCACATTAATTGGAGAAGC

B.1.351 (Beta variant):

TACTCAATAATTTGGGTGTGGACATTGCTGCTAATACTGTGATCTGGGA
CTACAAAAGAGATGCTCCAGCACATATATCTACTATTGGTGTTTGTTCTATGACTGACATAGCCAAGAAA
CCAACTGAAACGATTTGTGCACCACTCACTGTCTTTTTGATGGTAGAGTTGATGGTCAAGTAGACTTAT
TTAGAAATGCCCGTAATGGTGTTCTTATTACAGAAGGTAGTGTTAAAGGTTTACAACCATCTGTAGGTCC
CAAACAAGCTAGTCTTAATGGAGTCACATTAATTGGAGAAGC

UK- MW531680

TACTCAATAATTTGGGTGTGGACATTGCTGCTAATACTGTGATCTGGGA
CTACAAAAGAGATGCTCCAGCACATATATCTACTATTGGTGTTTGTTCTATGACTGACATAGCCAAGAAA
CCAACTGAAACGATTTGTGCACCACTCACTGTCTTTTTGATGGTAGAGTTGATGGTCAAGTAGACTTAT
TTAGAAATGCCCGTAATGGTGTTCTTATTACAGAAGGTAGTGTTAAAGGTTTACAACCATCTGTAGGTCC
CAAACAAGCTAGTCTTAATGGAGTCACATTAATTGGAGAAGC

B.1.1.7 (Alpha variant):

TACTCAATAATTTGGGTGTGGACATTGCTGCTAATACTGTGATCTGGGA
CTACAAAAGAGATGCTCCAGCACATATATCTACTATTGGTGTTTGTTCTATGACTGACATAGCCAAGAAA
CCAACTGAAACGATTTGTGCACCACTCACTGTCTTTTTGATGGTAGAGTTGATGGTCAAGTAGACTTAT
TTAGAAATGCCCGTAATGGTGTTCTTATTACAGAAGGTAGTGTTAAAGGTTTACAACCATCTGTAGGTCC
CAAACAAGCTAGTCTTAATGGAGTCACATTAATTGGAGAAGC

Italy- MT622321

TACTCAATAATTTGGGTGTGGACATTGCTGCTAATACTGTGATCTGGGA
CTACAAAAGAGATGCTCCAGCACATATATCTACTATTGGTGTTTGTTCTATGACTGACATAGCCAAGAAA
CCAACTGAAACGATTTGTGCACCACTCACTGTCTTTTTGATGGTAGAGTTGATGGTCAAGTAGACTTAT
TTAGAAATGCCCGTAATGGTGTTCTTATTACAGAAGGTAGTGTTAAAGGTTTACAACCATCTGTAGGTCC
CAAACAAGCTAGTCTTAATGGAGTCACATTAATTGGAGAAGC

USA- MT246470

TACTCAATAATTTGGGTGTGGACATTGCTGCTAATACTGTGATCTGGGA
CTACAAAAGAGATGCTCCAGCACATATATCTACTATTGGTGTTTGTTCTATGACTGACATAGCCAAGAAA
CCAACTGAAACGATTTGTGCACCACTCACTGTCTTTTTGATGGTAGAGTTGATGGTCAAGTAGACTTAT
TTAGAAATGCCCGTAATGGTGTTCTTATTACAGAAGGTAGTGTTAAAGGTTTACAACCATCTGTAGGTCC
CAAACAAGCTAGTCTTAATGGAGTCACATTAATTGGAGAAGC

Marocco- MT513758

TACTCAATAATTTGGGTGTGGACATTGCTGCTAATACTGTGATCTGGGA
CTACAAAAGAGATGCTCCAGCACATATATCTACTATTGGTGTTTGTTCTATGACTGACATAGCCAAGAAA
CCAACTGAAACGATTTGTGCACCACTCACTGTCTTTTTGATGGTAGAGTTGATGGTCAAGTAGACTTAT
TTAGAAATGCCCGTAATGGTGTTCTTATTACAGAAGGTAGTGTTAAAGGTTTACAACCATCTGTAGGTCC

CAAACAAGCTAGTCTTAATGGAGTCACATTAATTGGAGAAGC

B.1.427/B.1.429 (Epsilon variant)

TACTCAATAATTTGGGTGTGGACATTGCTGCTAATACTGTGATCTGGGA
CTACAAAAGAGATGCTCCAGCACATATATCTACTATTGGTGTTTGTTCTATGACTGACATAGCCAAGAAA
CCAACTGAAACGATTTGTGCACCACTCACTGTCTTTTTGATGGTAGAGTTGATGGTCAAGTAGACTTAT
TTAGAAATGCCCGTAATGGTGTTCTTATTACAGAAGGTAGTGTTAAAGGTTTACAACCATCTGTAGGTCC
CAAACAAGCTAGTCTTAATGGAGTCACATTAATTGGAGAAGC

Brazil- NC_045512.2

TACTCAATAATTTGGGTGTGGACATTGCTGCTAATACTGTGATCTGGGA
CTACAAAAGAGATGCTCCAGCACATATATCTACTATTGGTGTTTGTTCTATGACTGACATAGCCAAGAAA
CCAACTGAAACGATTTGTGCACCACTCACTGTCTTTTTGATGGTAGAGTTGATGGTCAAGTAGACTTAT
TTAGAAATGCCCGTAATGGTGTTCTTATTACAGAAGGTAGTGTTAAAGGTTTACAACCATCTGTAGGTCC
CAAACAAGCTAGTCTTAATGGAGTCACATTAATTGGAGAAGC

Omicron- OM570263

TACTCAATAATTTGGGTGTGGACATTGCTGCTAATACTGTGATCTGGGA
CTACAAAAGAGATGCTCCAGCACATATATCTACTATTGGTGTTTGTTCTATGACTGACATAGCCAAGAAA
CCAA**C**TGAAACGATTTGTGCACCACTCACTGTCTTTTTGATGGTAGAGTTGATGGTCAAGTAGACTTAT
TTAGAAATGCCCGTAATGGTGTTCTTATTACAGA**A**GGTAGTGTTAAAGGTTTACAACCATCTGTAGGTCC
CAAACAAGCTAGTCTTAATGGAGTCACATTAATTGGAGAAGC

XBB.1.5- OQ983940

TACTCAATAATTTGGGTGTGGACATTGCTGCTAATACTGTGATCTGGGA
CTACAAAAGAGATGCTCCAGCACATATATCTACTATTGGTGTTTGTTCTATGACTGACATAGCCAAGAAA
CCAA**T**TGAAACGATTTGTGCACCACTCACTGTCTTTTTGATGGTAGAGTTGATGGTCAAGTAGACTTAT
TTAGAAATGCCCGTAATGGTGTTCTTATTACAGA**G**GGTAGTGTTAAAGGTTTACAACCATCTGTAGGTCC
CAAACAAGCTAGTCTTAATGGAGTCACATTAATTGGAGAAGC

XBB.1.16- OR098785
TACTCAATAATTTGGGTGTGGACATTGCTGCTAATACTGTGATCTGGGA
CTACAAAAGAGATGCTCCAGCACATATATCTACTATTGGTGTTTGTTCTATGACTGACATAGCCAAGAAA
CCAA**T**TGAAACGATTTGTGCACCACTCACTGTCTTTTTGATGGTAGAGTTGATGGTCAAGTAGACTTAT
TTAGAAATGCCCGTAATGGTGTTCTTATTACAGA**G**GGTAGTGTTAAAGGTTTACAACCATCTGTAGGTCC
CAAACAAGCTAGTCTTAATGGAGTCACATTAATTGGAGAAGC

BA.2.86- PP405606
TACTCAATAATTTGGGTGTGGACATTGCTGCTAATACTGTGATCTGGGA
CTACAAAAGAGATGCTCCAGCACATATATCTACTATTGGTGTTTGTTCTATGACTGACATAGCCAAGAAA
CCAA**T**TGAAACGATTTGTGCACCACTCACTGTCTTTTTGATGGTAGAGTTGATGGTCAAGTAGACTTAT
TTAGAAATGCCCGTAATGGTGTTCTTATTACAGA**G**GGTAGTGTTAAAGGTTTACAACCATCTGTAGGTCC
CAAACAAGCTAGTCTTAATGGAGTCACATTAATTGGAGAAGC

JN.1- PP357841
TACTCAATAATTTGGGTGTGGACATTGCTGCTAATACTGTGATCTGGGA
CTACAAAAGAGATGCTCCAGCACATATATCTACTATTGGTGTTTGTTCTATGACTGACATAGCCAAGAAA
CCAA**T**TGAAACGATTTGTGCACCACTCACTGTCTTTTTGATGGTAGAGTTGATGGTCAAGTAGACTTAT
TTAGAAATGCCCGTAATGGTGTTCTTATTACAGA**G**GGTAGTGTTAAAGGTTTACAACCATCTGTAGGTCC
CAAACAAGCTAGTCTTAATGGAGTCACATTAATTGGAGAAGC

Supplementary Figure S6: Genome sequences of the predicted amplicons in various SARS-CoV-2 variants. The specific sequences of the predicted amplicons using the Set1 primer set, along with their translations depicted in Figure 2.

	Sample collection		Nucleic acid isolation		qPCR test	
**Materials**	Collection tube with regular buffer and a swab tube should be refrigerated and processed at the same day	Collection tube for saliva and another tube with lysis buffer. When mixed can be stored for two days at room temperature.	Sample decontaminated at 70C for 20 minutes. Seegene STARlet Equipment contains 300 ml sample plus 300 µl lysis buffer wash twice with 2 µl, elution with 100 µl from an expensive resin.	Directly handling a total of 2ml solution using a cheap resin. Concentration of the viral nucleic acid in 18 µl.	25 µl input sample into a total volume of 50 µl	3.2 µl input sample into a total volume of 10 µl
**Personnel**	Specialized person at a special facility to collect the swabs. Delivery of the samples to a special BSL3 lab.	patient self-collects the sample and mixes it with the lysis buffer, which also acts as a decontaminant. Subsequently, the sample is transported by a third party to a conventional laboratory	Specialized person at a special facility to denominate the samples in a special BSL3 lab.	Technician at a BSL2 laboratory		
**Added value of the RAPPID test which leads to a cheaper test**	Our method requires fewer resources, eliminates the need for refrigeration, specialized staff, or dedicated collection sites. Patients can self-test at home, reducing infection risks and encouraging greater testing uptake, ultimately lowering costs per sample.		Less reagents, significantly shorter assay, since the sample is already decontaminated (might lead to false negative due to RNA sensitivity). No need for a specialized BCL3 lab and no need for a specialized personnel, ultimately lowering costs per sample.		Less reagents and significantly shorter assay. Therefore, ultimately lowering costs per sample. Ability to process more sample at the same time .	
	SEEGENE Gold Standard Test	RAPID test				

Supplementary Figure S7: Cost-effectiveness and added value of the RAPID test (white boxes) compared to the gold standard SEEGENE test (gray boxes).
